# Some Reminiscences of St. Bartholomew's

**Published:** 1909-10-16

**Authors:** 


### FIFTY YEARS AGO.

### SOME REMINISCENCES OF ST. BARTHOLOMEW'S.

AN INTERVIEW WITH
SIR WILLIAM SELBY CHURCH, BART., K.C.V.O., F.R.S.

There can be nothing more interesting than the experiences of those who have trodden a path which the majority of us must still follow. The sum of experience, as Feizi, the philosopher of the great Akbar, remarks in one of the Vedic translations, is the greatest wisdom, and while this is true generally, every hospital worker must agree that it is even more particularly true in institutional matters. We want the opinions of men and women who have worked in medicine and in charitable institutions. Only such opinion counts, is worth listening to, worth acting upon. In the full confidence that readers of The Hospital are with us in this, that they share with us the feeling that much of this opinion is at present unexpressed, unvoiced, even unasked, and therefore sterile, it is our intention to give in these pages from time to time reports of special interviews with well-known leaders of thought and action in the realm of charitable work. We venture to think that such interviews will not only be interesting in themselves, but will contain much that is of value, and that may prove instructively useful to workers.

We have been fortunate enough to find, for the first victim of this inquisitorial method, the senior consulting physician of the oldest London hospital, Sir William Selby Church, who, in addition to his well-known work on many Royal Commissions, has had considerable experience of various London and provincial hospitals. Sir William was good enough to give our representative the benefit of his experience in matters of interest to hospital workers in the course of an interview last week.

### An Auto-Biographical Note.

"A man's life is always interesting, for the only thing that counts is personality." One need not go to the limit that Eduard Douwes Dekker reaches before accepting as proven the statement that forms the premiss of one of the syllogisms in the *Ideen*. What more natural, therefore, than to start the talk by trenching on the personal? Sir William will surely forgive the publication of his modest biographical note, which, meagre as it is in detail, and amplified as it can be by anyone who is acquainted with the share the President of the Royal Society of Medicine has taken in the development and the forward march of medicine in this country, will nevertheless prove interesting because it is in a sense autobiographical.

"My career in London," said Sir William, "started as a third year student, or what would nowadays be called so. I had done my preliminary work before I entered Bart's in 1862 as a dresser to Sir William Lawrence. In those days we went direct to the surgeon or physician and paid considerable fees to him for the privilege of dressing. Then I did a further and much longer term of work, this time as clinical clerk to Sir George Burrows, and Dr. Kirkes, and before I took my degree I also was an apprentice to Mr. Wood, the last resident apothecary in the hospital. My first connection with the staff dates back to the year 1866, when I was placed in charge of the cholera ward. Although we were outside the radius of the disease at its worst—that was more towards the east, and the London received the majority of cases—we had a good many cases. So far as I can remember only three of the attendants—all nurses—contracted the disease, and all three recovered. None of the staff got it. Since that time we have had no cholera cases in Bart's, with the exception of a few doubtful ones in the 'eighties. While serving in the cholera ward I acted for some months as resident apothecary at the hospital when Mr. Wood was ill, and I took over his work."

### Isolation Methods of the Sixties.

The subject of the cholera led to a talk on isolation, and Sir William contrasted the modern methods with those in vogue in his student days. "There were no isolation wards, of course, then," he said. "Take typhus for instance. We put our typhus cases in the general wards, and no attempt at isolation was made. I remember the dangers of this system only too well. During the very considerable epidemic of typhus in 1862 and 1863, or thereabouts, I and a colleague were working at the subject of typhus temperatures; I escaped, but he took the disease and died of it. We had very many typhus cases in those days—now you never see a case. During the five years 1860-1864 some 220 cases were admitted to Bart's, and in the epidemic year, '62 or '63, I don't exactly recollect which, there were 168 cases admitted, and on several occasions the medical wards had to be emptied on account of the spread of the disease. It was very much the same with the other infectious diseases—for instance, scarlet fever. Smallpox, however, was treated differently, and our smallpox cases were sent to the smallpox hospital, which dated, I believe, back to the eighteenth century. After the typhus epidemic isolation wards were started, mainly in consequence of the efforts of the medical staff. They were in charge of a special sister and nursing staff, all of whom had had the disease. That was a great advance."

"Bear in mind," he went on, earnestly, "that this mixing of typhus with general cases was not due to carelessness or ignorance. It was due very largely to the fact that we then considered that a few cases, spread in the general wards, did better, on the whole, than many cases massed together. The mortality among the typhus cases was less, and hospital authorities were really acting on what was considered the best advice of the time—on the recommendations, in fact, of a committee of the Royal College of Physicians."

### Progress in Treatment.

Touching the question of progress in treatment, Sir William, in reply to the request for an opinion, shook his head. "It is very difficult to say where progress has been the most apparent. There has been general progress, with great advances in some directions, and but little in others. Take typhus, for instance. So far as I know there has been no change made in the treatment of this disease by drugs—I am not talking now of the hygienic treatment, but purely of the drug treatment. If I were to say in what direction drug treatment has been most conspicuous—well, it is difficult. On the whole I think one would be justified in pointing to rheumatic fever. When I was a student, salicylate of soda was unknown in the treatment of acute rheumatic fever. Salicin, in the form of an infusion of the willow bark, had been known, though not often used, for years. Salicylic acid had been tried, but was not in frequent use, while the sodium salt was certainly not used in hospital practice. When it was tried, it became rapidly popular with us. During 1877 St. Bartholomew's Hospital used about 25 lbs. of it; the following year 45 lbs. were used, and in 1879 we used more than 70 lbs. Before that time the alkaline treatment was in vogue, and lemon juice was occasionally given. I do not think," Sir William continued, referring to the question of complications. "that the salicylate treatment has had much effect on the occurrence of endocarditis. There is not much difference in the number of cases that get endocarditis now and the number that we had under the old alkaline treatment, and the question of the incidence of endocarditis is largely one of age. I do not think that the salicylate treatment has been such a brilliant success as far as the sequelæ or complications are concerned; what it has done is that it has enormously diminished the patients' sufferings."

### Cold Baths as Antipyretics.

"Two other drugs I may mention," he proceeded, referring to a volume of the reports, "the iodides and bromides. I believe we have used them at Bart's since 1836, but I really do not know when they were first introduced. In 1864 we started using them in sensible quantities, and I think their use in that form marked a distinct advance in treatment. Then take cold bathing or sponging—I mean as an antipyretic. In my student days they were unknown. Cold was, of course, used as a means to reduce local temperature, but cold as a method of reducing the body temperature, was unpractised. Bear in mind that we were only just then starting to investigate the whole subject of temperature. There were no regular clinical thermometers in routine use. We had one or two in the wards, but they were curiosities almost, great big things, long and clumsy, or else the old forms of surface thermometers. The use of cold as an antipyretic seems to me to mark a distinct advance also in treatment. Currie had, of course, used the cold bath to reduce temperature more than a hundred years ago, but its use was by no means general. We made use of ordinary hot baths, hot air and steam baths, which were used in kidney cases, but nothing like cold baths or cold sponging and ice for cases of typhoid, pneumonia, or rheumatic fever. I can remember when there were no fixed baths at St. Bartholomew's. There used to be slipper baths, which were wheeled to the side of the bed, and as long as I was there I always kept such a bath handy. I think," Sir William remarked decisively, "these slipper baths ought to be in every ward."

### A Note on Baths and Bathing.

"I know there is a great fashion in baths nowadays. Everyone must have a fixed bath and a bathroom. Your houses will not let unless they have a bath-room, because people fancy they cannot do without them. They are a great saving of trouble, but for cleansing purposes are no better than the old slipper bath. In small private houses in rural districts as they are now introduced they waste far too much water, and increase all the difficulties of drainage without getting compensatory advantages. The old-fashioned bath, with comparatively little water, a big sponge, and soap, answered its purpose admirably, and it prevented waste. In a hospital ward it is different, but I would certainly have slipper baths as well. The modern fashion of taking the patient out of bed and carrying him to the other end of the ward before and after his bath does not appeal to me, and in most cases a bedside bath is preferable." Dealing with the water supply at hospitals, a matter which naturally arose out of his remarks anent the wastage of water in fixed baths, Sir William recalled the fact that St. Bartholomew's had been the first London hospital to make full use of the City water system. The New River Company was started in 1613, and on November 26, 1614, exactly a year after the works had been opened, there occurs in the hospital account books an item which shows that the first payment for water supplied to the hospital by the company had been made on that day. There used to be a well at the hospital, but this was closed, he thinks, after the first cholera epidemic. The present fountain stands on the site of the well, and there used to be a pump, shown in old prints of the hospital, which was finally removed shortly before Sir William went to the hospital.

### Hospital Abuse.

"Hospital abuse?" said Sir William, in answer to a remark about this important question. "Well, there has never been any hospital abuse, in the sense that is commonly understood, at Bart's. Our practice has always been—long before there was any hospital that possessed special almoners or inquiry officers—for the physician or surgeon at the out-patients' department to exercise his own discretion. If he thought that the patient was not one who should be treated at the hospital, or if he had any doubts about the financial position of the patient, he communicated with the office, and the case was at once investigated. A great deal of rubbish is talked about hospital abuse. The better-class of hospital patient has often paid as long as he is able to his medical attendant before he applies to the hospital. After he has received relief he is apt on the next occasion of illness to himself or in his family to go straight to the hospital."

"And the remedy is?"

"Ah! that is far too wide a question to discuss with you now. What I feel is this, that we have, as every hospital has, quantities of people who ought never to come to the hospital, but who ought to apply to the Poor Law infirmaries. It is with this class of patient that we have to deal, and it seems to me that the proper way to attack the problem is by means of provident dispensaries. Such dispensaries ought to be established, and the public ought to be interested in the matter. Personally, I think much more good would be done if people gave contributions towards the establishment of such institutions instead of giving large sums towards special hospitals as they are doing now. There is an association that has tried, and is trying, to arouse interest in these provident dispensaries. I take a great deal of interest in its work, and I think its efforts should receive better recognition than they do at present, for it is doing a very good work."

### A Point in Hospital Construction.

We turned the talk to hospital construction and hospitals generally, and Sir William remarked that in his student days there was no special septic ward at Bart's. Cases came in from the casualty station and were put in the general surgical wards. "We had cases of hospital gangrene and erysipelas, and you may imagine what the conditions were like. I believe it was not until the time of the establishment of the Asylums Board Hospitals that the surgeons obtained a septic ward—about 1880. Of course, there were always one or two rooms for offensive and foul cases." Sir William does not favour the special "dying room" which is so characteristic a feature of the newer Continental clinics. "There is no necessity for it," he argues. "It means the establishment of a special ward for those who are sentenced to die; and how are you going to divide your patients in the ordinary ward? What we want at Bart's, and what every hospital ought to have, are isolation rooms attached to each ward for the use of delirious or noisy patients. At present our noisy patients have to be taken right away from the ward and placed in a ward entirely removed from it, under a new sister and in charge of a new batch of nurses. This should not be. There should be a separate room attached to each ward for the use of such cases."

### Dietaries.

From hospital construction problems the conversation turned to hospital diets, and Sir William stated that one of the points of progress that had attracted his attention was the progress that had been made in the organisation and improvement of the hospital kitchen. The food formerly was less well looked after, and the surroundings were by no means ideal. He did not think there had been any marked changes in diet scales, with the exception of the change in the consumption of milk and alcoholic beverages. Forty or fifty years ago much more alcohol was consumed at Bart's than is at present used. Patients on full diet—dieta carnis—were allowed beer with their dinner and supper, a custom which is now no longer followed. This was an outstanding change which on the whole was satisfactory.

### Asepsis and Surgical Treatment.

Dealing with modern developments, Sir William paid the usual high tribute to antiseptics and asepsis, though, jealous of the reputation of his hospital, where at first Sir William Savory had looked askance at Listerian methods, he recalled the fact that we are apt to forget that surgical cleanliness is not a thing of 30 years only. "Mr. Callender," he remarked, "was most particular on this point. I well remember how he used to have his patients dressed with scrupulous attention to cleanliness. He had a special brush and basin or bottle for each patient in his wards, and these were never allowed to be interchanged. His results were remarkably good. At Bart's, I believe, the introduction of Listerian methods was due mainly to my colleague, the late Sir Thomas Smith, who went up to Edinburgh specially to get first-hand information. It was rapidly taken up, and there was really no opposition to it at any time, though Sir William Savory never fully gave in his adhesion to it."

The question of surgical treatment brought Sir William to speak of the relations between physicians and surgeons in hospital practice, and he pointed out how closely and cordially both had worked together at his own hospital. "Of course, that is seven years ago," he said with a smile, "but I do not think it has altered, though I do hear that our poor physicians now never get a case of appendicitis to cure. In my day we did cure a few without the help of the surgeon." Somehow the talk veered round to treatment at this point, and Sir William instanced, as a matter in which progress had been radical, the modern treatment of empyemata. He recalls the time "when it was a moot point whether empyemata should be opened or not." "Opinions differed, and the cases I saw did not prejudice me in favour of surgical treatment. Indeed, many of those that were opened were horribly distressing. The whole skin on the side round the wound not unusually sloughed in consequence of the decomposition of the discharging matter; the dressings could not be kept clean, the patient was in a deplorable condition, and sank rapidly. No wonder physicians would not readily consent to operation. Now all that is changed. There is, perhaps, no better illustration of the success that has attended antiseptic methods than this."

### Medical Students and Medical Education.

Finally, Sir William, being asked his opinion on the present-day medical student and his work, his relations with the hospital and the public, remarked that the subject was really too wide to permit of epitome within the compass of a concluding remark. "I think," he said, "the medical student has not changed very much; he is more or less as he was when I was a student, with the exception that he has got three times as much work to do and more examinations to pass. The student now depends far too much upon his teachers. When I was a student there were scarcely 30 teachers at Bart's—and I count in with these all the surgeons and physicians; now we have almost 80, while the number of students is much diminished. With regard to the hospitals, I am very strongly in favour of maintaining the voluntary principle so far as general hospitals, with medical schools attached, are concerned. In my opinion such a school should be entirely separate from the hospital to which it is attached. It should manage its own affairs, independent altogether of the hospital Board of Governors."

